# Circulatory microRNAs in helminthiases: Potent as diagnostics biomarker, its potential role and limitations

**DOI:** 10.3389/fvets.2022.1018872

**Published:** 2022-10-28

**Authors:** Hanif Ullah, Yali Tian, Safia Arbab, Ka Li, Muhammad Inayat Ullah Khan, Sajid Ur Rahman, Abdul Qadeer, Nehaz Muhammad, Suleman, Inam Ul Hassan

**Affiliations:** ^1^West China School of Nursing/West China Hospital, Sichuan University, Chengdu, China; ^2^Key Laboratory of Veterinary Pharmaceutical Development, Ministry of Agriculture, Key Laboratory of New Animal Drug Project of Gansu Province, Lanzhou Institute of Husbandry and Pharmaceutical Sciences, Chinese Academy of Agricultural Sciences, Lanzhou, China; ^3^State Key Laboratory of Biogeology and Environmental Geology, China University of Geosciences, Wuhan, China; ^4^Shanghai Veterinary Research Institute, Chinese Academy of Agricultural Sciences, Shanghai, China; ^5^Department of Food Science and Engineering, School of Agriculture and Biology, Shanghai Jiao Tong University, Shanghai, China; ^6^Department of Zoology, University of Swabi, Swabi, Pakistan; ^7^Department of Microbiology, Hazara University Manshera, Manshera, Pakistan

**Keywords:** circulatory microRNAs, biomarker, helminths, diagnosis, vesicle

## Abstract

Infections caused by helminths are responsible for severe public health problems and economic burden on continental scale. Well-timed and precise diagnosis of helminth infections is critical for taking by appropriate approaches for pathogen control. Circulating miRNAs are stable diagnostic tool for different diseases found in a variety of body fluid. As diagnostic biomarkers in infectious diseases, miRNAs detection in body fluids of helminth infected hosts is growing promptly. Uncovering miRNAs is a relatively new tool, used for early-stage detection of helminth infection from experimental or non-invasive clinical samples. miRNAs can be detected in body fluids such as serum, saliva, urine, and tissues of helminth infected host, mainly blood offering important benefits for diagnosis accurately. In this review, we discuss different characteristics of helminth parasite-derived circulating and EV miRNAs, supporting its potential uses in for helminth diagnosis and treatment efficiency.

## Introduction

Parasitic helminths, are classified into the phylum Platyhelminthes (including trematodes and cestodes) and the phylum Nematoda (nematodes) (1). The most common parasitic infection in humans is caused by soil-transmitted helminths (STHs), filarial worms (causing agents of onchocerciasis and lymphatic filariasis) and schistosomes. According to research, STHs such as *Trichuris trichiura* and *Ascaris lumbricoides*, and hookworms, infecting over 1.5 billion people globally (2). Schistosomiasis is a parasitic infection caused by the genus *Schistosoma*. Currently, it is existent in 78 countries, with 700 million people at risk of containing schistosomiasis (3–6). Foodborne trematodes are included *Clonorchis, Opisthorchis* and *Fasciola* which causes Clonorchiasis, Fascioliasis and Opisthorchiasis in human. According to WHO food borne trematodes are important causes of disability with an estimated annual total of 200,000 illnesses and more than 7,000 deaths per year (2). The World Health Organization (WHO) has already devised a strategy for controlling and eliminating helminth-related neglected tropical diseases. The most important helminth infection control strategy is mass drug administration (7).

MicroRNAs (miRNAs) are small, endogenously expressed, non-coding RNA transcripts with unique sequences that target mRNAs for post transcriptional regulation (8, 9). miRNAs have been identified in a wide range of organisms including helminths (10). Many miRNAs play important role in biological processes such as cell proliferation, growth, metabolism, and signal transduction (11). miRNA dys-regulation has also been linked to a variety of diseases, including non-communicable diseases like cancer, diabetes, kidney disease, and a variety of infectious diseases. Since last decade, the presence of miRNAs in various body fluids such as urine, serum, and plasma has been identified (12, 13). Circulating miRNAs are an effective and potential non-invasive biomarker in the pathology and prognosis of a wide range of morbidities, including infectious diseases. miRNA profiling in pathological conditions vs. healthy controls subjects can help predict disease stage (14). Extracellular vehicles (EVs), which are small membrane-bound secreted vesicles, play a role in many biological processes. EVs are present in all bodily fluids like saliva, urine, blood, serum and cerebrospinal fluid (15). In this review we demonstrated that circulating and EV derived miRNAs could be a new class of biomarkers for helminth detection and prognosis. We have briefly reviewed circulating miRNAs as diagnostic and prognostic markers the for helminth infections in the following sections.

## MicroRNAs in biological fluids

Several experimental studies have found extracellular/circulating miRNAs in biological fluids like plasma and serum (16), cerebrospinal fluid (17), saliva (18), breast milk (19), urine, tears, bronchial lavage, colostrum, peritoneal fluid, seminal fluid (20), and ovarian follicular fluid (21). Extracellular miRNAs exist in two populations in biological fluids. The first is found in vesicles like exosomes, micro-vesicles, and apoptotic bodies (22) while the second is associated with proteins, specifically AGO2 (23). There has been some debate over the relative abundance of these two populations. Researchers have discovered that the most of extracellular miRNAs are associated with AGO2 rather than micro-vesicles/exosomes (23), while other study discovered that extracellular miRNAs are predominantly existent in exosomes in serum and saliva of humans (18).

## Circulating miRNAs as diagnostic biomarkers

miRNA levels in the circulatory system may be altered by pathology in a relevant tissue. miRNAs, for example, may be inactively released during cell necrosis or vigorously secreted in vesicular structures during the course of liver disease (24). The small size and the establishment of a miRNA-protein complex, and their fusion into exosomes or other extracellular vesicles all contribute to their high stability in biofluids (EVs) (25). Because of the striking correlation between the status or progression of various diseases and miRNA dysregulation, circulating miRNAs have been regarded as promising targets for diagnostic biomarkers (25, 26). Circulating miRNAs have been studied for their diagnostic and predictive potential in a variety of diseases, such as cancer, liver damage, and viral infections (27, 28). A large number of miRNAs have been known in parasitic helminths, representing their importance in post-transcriptional regulation. During active helminthic infection, tissue and circulating miRNAs are dysregulated in mammalian hosts. Because helminth worm-derived miRNAs are released into the circulatory systems of mammalian hosts, they have the potential to be novel intervention targets and diagnostics for helminthiases. miRNAs are favorable therapeutic targets for parasitic helminth pathology, such as schistosome-induced liver fibrosis (10).

Furthermore, circulating miRNAs are highly stable under adverse conditions such as long-term storage, boiling, low or high pH, and multiple freeze-thaw cycles (13, 29). Previous study detected *Mycobacterium avium* subspecies paratuberculosis in bovine serum samples, and revealed that the circulating miRNA profile of samples stored for 10–15 years at −20°C was quite similar to that of fresh serum samples (stored for <1 year at −80°C) (30). Another research examined the miRNA stability in serum and plasma from healthy dogs after varying amounts of time at room temperature, and the authors demonstrated that miRNAs were highly stable when stored at room temperature for 1 h but not for 24 h (31). Because of the above-mentioned characteristics of circulating miRNAs, they have unique interest of researchers as potential diagnostic biomarkers for a variety of diseases.

## Role of microRNA in varies disease

miRNAs play a variety of biological roles in various processes within living organisms (32, 33). i.e., angiogenesis, metastasis, invasion, growth, differentiation, and apoptosis (34, 35). miRNAs play critical role in the initiation and advancement of various carcinomas (36, 37). A wide range of biomarkers, including microRNAs (miRNAs), tPA, and von Willebrand factor, could be used as diagnostic and therapeutic biomarkers in stroke therapy (32). Circulating miRNAs are a type of miRNA that can be used as biomarkers for prognosis, diagnosis, and treatment (38). In serum and plasma samples, intact, cell-free miRNAs that are resistant to nuclease digestion have recently been discovered. These miRNAs are circulating and quantifiable, suggesting that they could be used as noninvasive, sensitive diagnostic or progression biomarkers in a variety of diseases. MiRNAs in circulation as biomarkers for monitoring therapy response and distinguishing normal from breast cancer cells (39). A study found that circulating miR-155 levels were higher in the serum of breast cancer patients (40). According to research, miR-155 could be a encouraging biomarker for breast cancer diagnosis, as well as a predictor of treatment response (41). Circulating miRNAs in plasma and serum come into direct contact with blood cells. In 2008, the initial report on the changed levels of circulating miRNAs in hematologic cancers was conducted in patients with diffuse large B cell lymphoma (DLBCL) (12). In this study, miR-155, miR-21 and miR-210 were found to be significantly higher in cancer patients' sera than in healthy control subjects (42). Patients with chronic lymphocytic leukemia (CLL), had significantly altered circulating miRNAs (43). Seven plasma miRNAs (miR-223, miR-150, miR-92a, miR-19b, miR-320, miR-17, and miR-484,) were reported to be extremely abundant in all CLL samples tested. Furthermore, changes in circulating miR-195 or miR-20a levels were found to be the best classifiers for distinguishing CLL patients from healthy control subjects (43). Circulating miRNAs were also found in patients with liver cancers; six serum miRNAs (miR-92a, miR-1, miR-375, miR-25, miR-206, and let-7f) were found to be significantly up - regulated in hepatocellular carcinoma (HCC) samples compared to healthy controls. Furthermore, three of the six miRNAs (miR-25, let-7f, and miR-375) were shown to be capable of clearly distinguishing HCC cases from healthy control subjects (44). In a recent study, over 20 miRNAs (including miR-92a) were found to be overexpressed in the sera of HCC patients (44).

A majority of studies have shown that their dysregulation can lead to the onset and development of various diseases such as stroke, diabetes, inflammatory diseases and cardiovascular disease (45, 46). Sun et al. (47) discovered that miR-124 could be used as diagnostic and therapeutic biomarkers in stroke patients, as it is released from brain tissue plays an important role in stroke. Other miRNAs that can be used as diagnostic biomarkers in stroke patients include miR-320. miR-320 expression is significantly reduced in stroke patients. Down-regulation of this miRNA has been shown to initiate anti-apoptotic processes in CNS tissue (48). Various circulating miRNAs were tested as diagnostic biomarkers in stroke patients by Sepramaniam et al. their findings show that circulating miRNAs such as miR-27a, miR-125b-2, miR-488, miR-422a, and miR-627 express differently in stroke patients than in healthy subjects. They revealed that these circulating miRNAs were highly expressed in stroke patients (49). Several scientists have found that miRNAs play important roles in diabetes pathogenesis (45, 50). Deregulation of several miRNAs, including miR-21, miR-15, miR-144, miR-192 and miR-150 has been linked to diabetes pathogenesis (50).

miRNAs can also be used for diagnosis of protozoan infections for humans such as Malaria, Leishmaniasis, Toxoplasmosis, and Trypanosomiasis (51, 52). In malaria infection, miRNAs facilitate invade and grow of the parasite in red blood cells via escape from immune responses and defect of opsonization by circulating macrophages (53). Chamnanchanunt et al. (54) showed lower levels of hsa-miR-451 and hsa-miR-16 in plasma and RBCs of *Plasmodium vivax* human patients than non-infected subjects. Baro et al. (55) demonstrated hsa-miR-221, hsamiR-222, hsa-miR-24, and hsa-miR-19 were decreased in *P. vivax* patient's RBCs. Nahid et al. (56) showed *Leishmania major* could change miRNA levels in macrophages after infection of the cells with the parasite; it was identified that downregulation of hsa-let-7a expression was in 48 h after infection. In a study by Geraci et al., next-generation sequencing is applied to study the levels of miRNAs in human monocyte derived dendritic cells (DC) and macrophages (MP) infected with either *L. major* or *L. donovani* parasites. The results of the study showed that *L. donovani* has a prominent role in the upregulation of hsa-miR-21 and hsamiR-146b-5p and proteins involved in the TGF-βsignaling pathway, such as SMAD7 and TRAF6 (57).

Parasitic helminths, including trematodes, tapeworms, cestodes, and nematodes, are the most common human infectious agents in developing countries (52, 58). Approximately 1 billion people live in the slums of the continents Africa, Asia, and America, infected with one or more worms (58, 59). Therefore, identification, diagnosis, treatment, and control of worm infections should be among the most essential actions in these areas (60).

## MicroRNAs; circulating miRNAs as diagnostic biomarkers for helminths infection

The emergence of parasitic helminth genome sequence data has paved the way for the identification of miRNA sequences using both experimental and computational approaches. Some miRNA sequences are conserved across species, which can be used to identify potential miRNAs through artificial intelligence (61). Documentation of miRNA populations in at least 36 parasitic helminth species (8 cestodes, 11 trematodes, and 17 nematodes, including two plant parasitic nematodes, *Bursaphelenchus xylophilus* and *Globodera pallida*) has been accomplished using miRNA prediction tools such as miRDeep2, mireap, and miR analyzer, or through homologous mapping. 616 pre-miRNAs from *Echinococcus multilocularis, Schistosoma mansoni, Ascaris suum, Echinococcus granulosus, Brugia malayi*, and *Haemonchus contortus* have been annotated in miRBase (10). Lin-4, the first reported nematode miRNA, was discovered in free-living *Caenorhabditis elegans* 20 years ago (62). However, the first detailed investigation of parasitic nematode miRNAs was published in 2010 for a filarial nematode *B. malayi* (63), and later extended to include other parasitic nematodes (e.g., *Trichinella spiralis, H. contortus*, and *A. suum*). MiRNAs have been discovered in *S. japonicum*, a trematode (64) and *S. mansoni* (65), *Fasciola gigantica, F. hepatica* (66), and *Eurytrema pancreaticum* (67). Although the vast majority of miRNAs are endogenous, only a small number enter the blood circulation and circulate throughout the body (68). These cell-free miRNAs have been found in urine, blood plasma, and serum (25, 69). miRNAs derived from parasitic helminths are perceptible in the biofluids of their mammalian hosts and can be used to diagnose infection, even when the parasitic species do not reside in the host's circulatory system (14). The presence of parasite-derived miRNAs in the serum/plasma of the definitive host with helminth infection has increased interest in testing worm-derived miRNAs as specific biomarkers for specific helminthic infections (10). Circulating miRNAs have recently been proposed as a new class of biomarkers for diagnosing helminth infection.

Several studies on trematodes have been conducted for identification of miRNA. Schistosomiasisis a neglected tropical parasitic disease associated with severe pathology, Mortality and economic loss worldwide. Cheng et al. reported schistosome-specific five miRNAs (Bantam, miR-3479, sja-miR-8185, miR-3096 and miR-10) in rabbits infested with *S. japonicum*. Four of these five miRNAs were found to be significantly abundant in the plasma of mice infected with *S. japonicum*, implying that these miRNAs could be used as a biomarker for schistosomiasis diagnosis (70). A six miRNA candidates were validated with serum samples from a human cohort in a schistosomiasis endemic area of the Philippines, which showed that two parasite derived miRNAs (sja-miR-2b-5p and sja-miR-2c-5p) could be detected in infected individuals with a moderate diagnostic performance (68). In low-dose cercarial infection models, it was confirmed that sja-miR-277 and sja-miR-3479-3p, but not sja-bantam, could be reliably detected in mouse serum, regardless of mouse strain. We were able to demonstrate a significant correlation between sja-miR-3479-3p, sja-miR-277, levels in serum and both the degree of fibrosis and egg burden in the liver using these precise methods (71). In low doses, these parasite-derived circulating miRNAs demonstrated additional potential as biomarkers for early detection of *S. japonicum* infection. According to new evidence, altered host miRNAs can be used to diagnose *S. japonicum* liver infection. Elevated levels of miR-223 in serum samples from human, rabbit, buffalo, and mouse hosts could be used to detect *S. japonicum* infection (72). In BALB/c mice infected with *S. japonicum*, serum levels of mmu-miR-21, mmu-miR-122 and mmumiR-34a were significantly higher (73). Furthermore, several additional studies demonstrated that parasite-derived miRNAs (miR-3479-3p, miR-277, and bantam) could detect individuals infected with *S. haematobium* or *S. mansoni* using real time qPCR in low and high infection intensity sites (74, 75). Circulating miRNAs were also identified in buffaloes infected with *F. gigantica*, a tropical liver fluke and the cause of fascioliasis; four worm-specific miRNAs, fgi-miR-87, fgi-miR-71, fgi-miR-124 and, the novel miR-1, were identified in the sera of infected animals by deep sequencing (76).

miRNAs derived from various nematode species have been found circulating in host tissue and the circulatory system. Tritten et al. reported the existence of miRNA (nematode-derived) candidates in the plasma of dogs infected with *Dirofilaria immitis*, a filarial heartworm, using a veterinary model (77). Similarly, in two independent studies, a panel of *Onchocerca*-derived mature miRNAs including miR-100a/c/d, miR-81, and miR-71 were identified in the nodule fluid and the plasma from bovine infected with *O. ochengi* (78). Furthermore, miRNA candidates of potential nematode origin, such as miR-36, miR-100a/d, miR-92, and lin-4, were discovered in the plasma of baboons infected with the ‘eye worm *Loa loa* (78). miRNAs derived from nematodes, such as lin-4, miR-100a/d, and miR-71, were also discovered in the serum or plasma of people infected with *O. volvulus*, the causative agent of onchocerciasis (79). Silakit et al. revealed that miR-192 levels were significantly higher in cholangiocarcinoma (CCA) patients' serum than in healthy donors. Furthermore, has-miR-192 expression levels were found to be elevated in both patient-derived CCA liver tissues and *Opisthorchis viverrini*-induced CCA liver tissues from a hamster model (80). Strikingly, plasma miRNA profiling disclosed that eight miRNAs including hsa-miR-885-5p, hsa-miR-505-3p, hsa-miR-483-5p, hsa-miR-92b-3p, hsa-miR-874, has-miR-1307-3, hsa-miR-1275, and hsamiR-320b, associated with Intrahepatic cholangiocarcinoma (ICC) are induced by *O. viverrini*, developing the basis of a circulating miRNA-based biomarker panel for ICC (81). A study evaluating the circulating miRNAs released by *Angiostrongylus cantonensis* (the cause of eosinophilic meningoencephalitis) as potential biomarkers of infection, found that the level of aca-miR-146a in serum was significantly higher in *A. cantonensis*-infected mice compared with uninfected control animals (82).

Cestode-derived miRNA, emu-miR-277 and emu-miR-10 were found in the serum of all *E. multilocularis* infected mice. Furthermore, gene expression of mmu-miR-146a-5p, mmu-miR-107-3p, mmu-miR-103-3p, mmu-miR-339-5p, and mmu-miR-21a-3p was increased in infected mice sera while mmu-miR-222-3p was decreased (83). The study reported by Alizadeh et al. (84) that the levels of two circulating worm-specific miRNAs (egr-miR-71 and egr-let-7) were detectable in the plasma of patients infected with *E. granulosus* compared with uninfected individuals. Here in Table 1, we summarize some circulating miRNAs of helminth infection.

**Table 1:** Circulatory miRNAs detected from infected hosts.

**Pathogens**	**Circulating miRNA**	**Sample**	**References**
*Loa Loa*	miR-36, miR-92, lin-4, miR-100a/d	Baboon plasma	(79)
*Onchocerca. ochengi*	miR-100a, miR-81	Cow plasma	(79)
*O. ochengi*	miR-71, bantam –a/b/c, miR-100a/c/d miR-86-5p,	Cattle nodule fluids	(78)
*S. japonicum*	miR-150-5p, miR-146a-5p and let-7d-5p, let-7a-5p	Mouse/human serum	(85)
*O. volvulus*	miR-87-3p, miR-100a/d, miR-71, bantam-a, and Lin-4,	Human plasma and serum	(78)
*O. volvulus*	ov-miR71-23nt, ov miR-100d, ov-bantam-a, ov miR71-22nt, bma-miR-71, bma-miR-236-1, and ov-miR-87-3p	human serum	(86)
*Litomosoides sigmodontis*	miR-71, miR-86, bantam-a/b, miR-100a/b/c,	Mouse serum	(87)
*O. viverrini*	has-miR-192	Human Serum	(80)
*D. immitis*	miR-34, miR-71, miR-228 and miR-100a/c/d,	Dog plasma	(77)
*S. mansoni*	sma-bantam, sma-miR-3479-3p and Sma-miR-277,	Mouse/human serum	(75)
*S japonicum*	sja- bantam, sja-miR-3479-3p and Sja-miR-277,	Mouse serum/ rabbit serum	(70, 71)
*O. viverrini*	hsa-miR-1275, hsa-miR-320b, hsa-miR-483-5p, hsa-miR-505-3p, hsa-miR-92b-3p, hsa-miR-1307-3, hsa-miR-874, and hsa-miR-885-5p	Human Plasma	(81)
*S. japonicum*	mmu-miR-134-5p, [mmu-let-7b-3p, mmu-miR-1194, mmu-miR-1981-3p, mmu-miR-210-5p], [mmu-miR-542-3p and mmu-miR-92a-2-5p]	Mouse Plasma	(88)
*S. japonicum*	mmu-miR-21, mmu-miR-34a and mmu-miR-122,	Mouse/human Serum	(71)
*S. japonicum*	sja-miR-2b-5p and sja-miR-2c-5p	human serum	(68)
*E. multilocularis*	emu-miR-227 and emu-miR-10	Mice serum	(83)
*S. japonicum*	miR-223	Human, Rabbit, Buffalo and Mouse serum	(72)

## MicroRNAs detect from extracellular vesicle

Extracellular vehicles (EVs), which are small membrane-bound secreted vesicles, play a role in many biological processes (89). According to mounting evidence, exosomes and other extracellular structures (e.g., microvesicles) containing specific proteins, miRNA or miRNA-like molecules are actively released by parasites (90). EVs help in pathogens spread and play a regulatory role in the host immune system. Exosomes have been isolated from several body fluids, including serum, urine and saliva (91). EV molecules as biomarkers for the diagnosis and prognosis of diseases (92). However, there is still much to be explored regarding the applications of helminth-derived EVs, such as their potential in diagnosis, as therapeutics (93). Recent studies have identified that EVs isolated and characterized from helminth parasites such as proteomic investigation of *S. mansoni* EVs revealed that these are potential vaccine candidates (94); exosomes from *S. japonicum* induce M1-type immune-activity in macrophages *in vitro* (95), *Echinostoma caproni* and *F. hepatica* EVs are internalized into gut host cells (96), and miRNAs are related with *F. hepatica* EVs (97). Exosome components are being studied for their potential value as a source of biomarkers for cancer and other diseases (98). The discovery of *S. mansoni* EVs and the characterization of their cargo via combinatorial protein/sncRNA characterization points to the identification of an important new participant in the complex biology underlying schistosome/host interactions. Further research into the function and stability of intra- and extra-vesicular sncRNA components, as well as the role of these *S. mansoni* EVs, could lead to the development of novel schistosomiasis diagnostics or interventions (94). EVs secreted by *O. viverrini* have been shown to induce a tumorigenic phenotype and proinflammatory in human cholangiocytes (99). Notably, Bernal et al. (100) were the first to discover miRNAs in exosomes secreted by *Dicrocoelium dendriticum*, a ruminant trematode. Ancarola et al. reported seven unique miRNAs (miR-190-5p, let-7-5p, miR-4989-3p, miR-61-3p, miR-219-5p, miR-71-5p, and miR-277-3p), in *Taenia crassiceps* vesicles, but only let-7-5p was described in *Mesocestoides corti* EVs. These microRNAs are not only useful for basic cestode biology, but also for the rational search for new diagnostic targets (101). According to Zhu et al. (102) miRNA-containing EVs can be released by *S. japonicum* eggs, and the EVs can transfer their cargo to recipient cells *in vitro*. Another recent study found miRNA, Y RNA, and an Argonaute protein in exosomes secreted by *Heligmosomoides polygyrus*, a rodent parasitic nematode (87). It has been discovered that these *H. polygyrus* miRNA signatures may play a role in modulating host innate immunity (87). Furthermore, miRNAs enriched in EVs secreted by *F. hepatica* have been linked to immune-regulatory function, tissue growth, and cancer (97). Here we summarize some miRNAs detected from extracellular vesicle in Table 2.

**Table 2:** MicroRNA detect from extracellular vesicle.

**Pathogens**	**miRNAs detected in extracellular vesicle**	**Sample**	**References**
*S. japonicum*	*miR-125b* and *bantam*	Worm	(103)
*F. hepatica*	miR-1, miR-125b, miR-71a, miR-190 and miR-87	Exosome like vesicle from adult worms	(97)
*D. dendriticum*	miR61, miR-190, miR-71, miR-2a-3p and let-7	Exosome from adult worms	(100)
*Heligmosomoides polygyrus*	miR-7, miR-87, bantam, Lin-4, miR-71 and miR-100	Adult worms' Secretory product	(87)
*Trichuris suis*	miRNA-size RNA	L1-stage larvae Secretory product	(104)
*S. mansoni*	Sma-miR-new-204, Sma-miR-new-176, Sma-miR-new-271, Sma-miR-new- 215, and Sma-miR-new-270	Schistosomula	(94)
*T. crassiceps*	miR-71-5p, miR-219-5p, miR-4989-3p, miR-277-3p, miR-61-3p, miR-190-5p, and let-7-5p	Adult worm	(101)
*M. corti*	let-7-5p	Adult worm	(101)
*S. japonicum*	sja-miR-71a, sja-miR-36-3p, sja-bantam, sja-miR-2a-3p, and sja-miR-10-3p	Egg EVs	(102)

## Limitation of circulatory miRNA biomarker

As we discussed above, circulating miRNAs are becoming potential non-invasive biomarkers for the diagnosis of helminths infection. Despite there are still challenges to overcome before clinical application. Both limitations and potentials of miRNAs should be taken into account when diagnosing helminth infections. Studies had shown that although the use of biomarkers was a practical and unique idea in diagnosing diseases, the necessary and sufficient conditions for its use must be provided, including storage conditions of samples correct selection of primers and type of miRNAs, correct data analysis, and the use of experienced people (72, 105). miRNAs can be detected using various specific and sensitive approaches, including Northern blot Analysis (106), *in situ* hybridization (107), real-time PCR (108), miRNA microarray (109) and next-generation sequencing (110), and some of them are already used as diagnostic or prognostic markers. This demonstrates their utility in both clinical and personalized medicine. However, it has several limitations, such as the high cost of the instrumentation required for thermal cycling and signal detection, and the need for well-equipped laboratories (7). miRNAs biomarkers has the potential to address the shortcomings of other diagnostic tests, including cross-corrections, false negatives, low sensitivity, and specificity (111, 112). In addition, whereas the costs for developing diagnostic tools targeting circulating miRNA and EV components such as sncRNAs and proteins are high, expenditure could be reduced substantially if multiplex or high-through-put assays targeting multiple helminths and/or non-helminth pathogens are developed for simultaneous application (113).

## Conclusion

In the current situation the helminth infection has become more prevalent, numerous strategies with high sensitivity and specificity have been developed. The diagnosis of helminth can be using diagnostic methods like PCR, qPCR, serology, and next-generation sequencing that target the miRNA and worm EVs. The detection of miRNA may be a feasible substitute to the in-use conventional measures which involve the use of multiple blood, serum, saliva, urine samples for the detection of helminth infection in any stages. Circulating miRNAs have the potential to serve as non-invasive biomarkers for the early detection of helminth infection due to their accessibility and long-term stability. Extracellular/circulating miRNAs are now recognized as important players in intercellular communication as well as biomarkers for diseases. Further research on circulating miRNA profiles would broaden helminth biomarker research and enable the development of diagnostic strategies and examinations based on a sensitive and simple test.

## Author contributions

HU collected the data and prepared draft. All authors have shared in all aspects and read and agreed to the published version of the manuscript.

## Conflict of interest

The authors declare that the research was conducted in the absence of any commercial or financial relationships that could be construed as a potential conflict of interest.

## Publisher's note

All claims expressed in this article are solely those of the authors and do not necessarily represent those of their affiliated organizations, or those of the publisher, the editors and the reviewers. Any product that may be evaluated in this article, or claim that may be made by its manufacturer, is not guaranteed or endorsed by the publisher.

## References

[1] Kaliappan SP George S Francis MR Kattula D Sarkar R Minz S. Prevalence and clustering of soil-transmitted helminth infections in a tribal area in southern India. Trop Med Int Health. (2013) 18:1452–62. 10.1111/tmi.12205 24237860 PMC3923153

[2] WHO. World Health Organization Fact-Sheets. Geneva: WHO (2019). Available online at: http://www.who.int/news-room/fact-sheets/detail/schistosomiasis (accessed 2019)

[3] Ullah H Arbab S Khan MIU Li K Muhammad N Qadeer A. Circulating cell-free mitochondrial DNA fragment: a possible marker for early detection of *S. japonicum. Infect Genet Evol*. (2021) 88:104683. 10.1016/j.meegid.2020.104683 33348056

[4] Ullah H Qadeer A Giri BR. Detection of circulating cell-free DNA to diagnose *S. japonicum* infection. Acta Trop. (2021) 211:105604. 10.1016/j.actatropica.2020.105604 32598919

[5] Ullah H Arbab S Li K Khan MIU Qadeer A Muhammad N. Schistosomiasis related circulating cell-free DNA: a useful biomarker in diagnostics. Mol Biochem Parasitol. (2022) 251:111495. 10.1016/j.molbiopara.2022.111495 35835258

[6] Qadeer A Giri BR Ullah H Cheng G. Transcriptional profiles of genes potentially involved in extracellular vesicle biogenesis in *S. japonicum. Acta Trop*. (2021) 217:105851. 10.1016/j.actatropica.2021.105851 33524382

[7] Ullah H Qadeer A Rashid M Rashid MI Cheng G. Recent advances in nucleic acid-based methods for detection of helminth infections and the perspective of biosensors for future development. Parasitology. (2020) 147:383–92. 10.1017/S0031182019001665 31840627 PMC10317639

[8] Baltimore D Boldin MP. O'connell RM, Rao DS, Taganov KD. MicroRNAs: new regulators of immune cell development and function. Nat Immunol. (2008) 9:839. 10.1038/ni.f.209 18645592

[9] Sonkoly E Pivarcsi A. Advances in microRNAs: implications for immunity and inflammatory diseases. J Cell Mol Med. (2009) 13:24–38. 10.1111/j.1582-4934.2008.00534.x 19175698 PMC3823034

[10] Cai P Gobert GN McManus DP. MicroRNAs in parasitic helminthiases: current status and future perspectives. Trends Parasitol. (2016) 32:71–86. 10.1016/j.pt.2015.09.003 26489492

[11] Cheng G Danquah M Mahato RI. MicroRNAs as therapeutic targets for cancer. Pharm Perspect Cancer Therap. (2009) 9:441–74. 10.1007/978-1-4419-0131-6_14

[12] Lawrie CH Gal S Dunlop HM Pushkaran B Liggins AP Pulford K. Detection of elevated levels of tumour-associated microRNAs in serum of patients with diffuse large B-cell lymphoma. Br J Haematol. (2008) 141:672–5. 10.1111/j.1365-2141.2008.07077.x 18318758

[13] Mitchell PS Parkin RK Kroh EM Fritz BR Wyman SK Pogosova-Agadjanyan EL. Circulating microRNAs as stable blood-based markers for cancer detection. PNAS. (2008) 105:10513–8. 10.1073/pnas.0804549105 18663219 PMC2492472

[14] Ghalehnoei H Bagheri A Fakhar M Mishan MA. Circulatory microRNAs: promising non-invasive prognostic and diagnostic biomarkers for parasitic infections. Eur J Clin Microbiol Infect Dis. (2019) 39:1–8. 10.1007/s10096-019-03715-8 31617024

[15] Karin-Kujundzic V Marija Sola I Predavec N Potkonjak A Somen E Mioc P. Novel epigenetic biomarkers in pregnancy-related disorders and cancers. Cells. (2019) 8:1459. 10.3390/cells8111459 31752198 PMC6912400

[16] Chen X Ba Y Ma L Cai X Yin Y Wang K. Characterization of microRNAs in serum: a novel class of biomarkers for diagnosis of cancer and other diseases. Cell Res. (2008) 18:997. 10.1038/cr.2008.282 18766170

[17] Cogswell JP Ward J Taylor IA Waters M Shi Y Cannon B. Identification of miRNA changes in Alzheimer's disease brain and CSF yields putative biomarkers and insights into disease pathways. J Alzheimer's Dis. (2008) 14:27–41. 10.3233/JAD-2008-14103 18525125

[18] Gallo A Tandon M Alevizos I Illei GG. The majority of microRNAs detectable in serum and saliva is concentrated in exosomes. PLoS ONE. (2012) 7:e30679. 10.1371/journal.pone.0030679 22427800 PMC3302865

[19] Zhou Q Li M Wang X Li Q Wang T Zhu Q. Immune-related microRNAs are abundant in breast milk exosomes. Int J Biol Sci. (2012) 8:118. 10.7150/ijbs.8.118 22211110 PMC3248653

[20] Weber JA Baxter DH Zhang S Huang DY Huang KH Lee MJ. The microRNA spectrum in 12 body fluids. Clin Chem. (2010) 56:1733–41. 10.1373/clinchem.2010.147405 20847327 PMC4846276

[21] da Silveira JC Veeramachaneni DR Winger QA Carnevale EM Bouma GJ. Cell-secreted vesicles in equine ovarian follicular fluid contain miRNAs and proteins: a possible new form of cell communication within the ovarian follicle. Biol Reprod. (2012) 86:71. 10.1095/biolreprod.111.093252 22116803

[22] Iftikhar H Carney GE. Evidence and potential in vivo functions for biofluid miRNAs: from expression profiling to functional testing: potential roles of extracellular miRNAs as indicators of physiological change and as agents of intercellular information exchange. Bioessays. (2016) 38:367–78. 10.1002/bies.201500130 26934338

[23] Turchinovich A Weiz L Langheinz A Burwinkel B. Characterization of extracellular circulating microRNA. Nucl Acids Res. (2011) 39:7223–33. 10.1093/nar/gkr254 21609964 PMC3167594

[24] Roderburg C Luedde T. Circulating microRNAs as markers of liver inflammation, fibrosis and cancer. J Hepatol. (2014) 61:1434–7. 10.1016/j.jhep.2014.07.017 25306489

[25] Szabo G Bala S. MicroRNAs in liver disease. Nat Rev Gastroenterol Hepatol. (2013) 10:542. 10.1038/nrgastro.2013.87 23689081 PMC4091636

[26] Schwarzenbach H Nishida N Calin GA Pantel K. Clinical relevance of circulating cell-free microRNAs in cancer. Nat Rev Clin Oncol. (2014) 11:145. 10.1038/nrclinonc.2014.5 24492836

[27] Starkey Lewis P Merz M Couttet P Grenet O Dear J Antoine D. Serum microRNA biomarkers for drug-induced liver injury. Clin Pharmacol Ther. (2012) 92:291–3. 10.1038/clpt.2012.101 22828715

[28] Kosaka N Iguchi H Ochiya T. Circulating microRNA in body fluid: a new potential biomarker for cancer diagnosis and prognosis. Cancer Sci. (2010) 101:2087–92. 10.1111/j.1349-7006.2010.01650.x 20624164 PMC11159200

[29] Brase JC Wuttig D Kuner R Sültmann H. Serum microRNAs as non-invasive biomarkers for cancer. Mol Cancer. (2010) 9:306. 10.1186/1476-4598-9-306 21110877 PMC3002336

[30] Shaughnessy RG Farrell D Riepema K Bakker D Gordon SV. Analysis of biobanked serum from a *Mycobacterium avium* subsp paratuberculosis bovine infection model confirms the remarkable stability of circulating miRNA profiles and defines a bovine serum miRNA repertoire. PLoS ONE. (2015) 10:e0145089. 10.1371/journal.pone.0145089 26675426 PMC4682966

[31] Enelund L. N Nielsen L, Cirera S. Evaluation of microRNA stability in plasma and serum from healthy dogs. Microrna. (2017) 6:42–52. 10.2174/2211536606666170113124114 28088915

[32] Mirzaei H. Stroke in women: risk factors and clinical biomarkers. J Cell Biochem. (2017) 118:4191–202. 10.1002/jcb.26130 28498508

[33] Rashidi B Hoseini Z Sahebkar A Mirzaei H. Anti-atherosclerotic effects of vitamins D and E in suppression of atherogenesis. J Cell Physiol. (2017) 232:2968–76. 10.1002/jcp.25738 27966778

[34] Fathullahzadeh S Mirzaei H Honardoost M Sahebkar A Salehi M. Circulating microRNA-192 as a diagnostic biomarker in human chronic lymphocytic leukemia. Cancer Gene Ther. (2016) 23:327–32. 10.1038/cgt.2016.34 27659777

[35] Mirzaei H Khataminfar S Mohammadparast S Shahid Sales S Maftouh M. Circulating microRNAs as potential diagnostic biomarkers and therapeutic targets in gastric cancer: current status and future perspectives. Curr Med Chem. (2016) 23:4135–50. 10.2174/0929867323666160818093854 27538692

[36] Mohammadi M Goodarzi M Jaafari M Mirzaei H Mirzaei H. Circulating microRNA: a new candidate for diagnostic biomarker in neuroblastoma. Cancer Gene Ther. (2016) 23:371–2. 10.1038/cgt.2016.45 27740613

[37] Mirzaei H Sahebkar A Jaafari M Hadjati J Javanmard S. Mirzaei H. PiggyBac as a novel vector in cancer gene therapy: current perspective. Cancer Gene Ther. (2016) 23:45–7. 10.1038/cgt.2015.68 26742580

[38] Reza Mirzaei H Sahebkar A Mohammadi M Yari R Salehi H. Hasan Jafari M. Circulating microRNAs in hepatocellular carcinoma: potential diagnostic and prognostic biomarkers. Curr Pharm Des. (2016) 22:5257–69. 10.2174/1381612822666160303110838 26935703

[39] Zhu W Qin W Atasoy U Sauter ER. Circulating microRNAs in breast cancer and healthy subjects. BMC Res Notes. (2009) 2:89. 10.1186/1756-0500-2-89 19454029 PMC2694820

[40] Roth C Rack B Müller V Janni W Pantel K Schwarzenbach H. Circulating microRNAs as blood-based markers for patients with primary and metastatic breast cancer. Breast Cancer Res. (2010) 12:R90. 10.1186/bcr2766 21047409 PMC3046429

[41] Heneghan HM Miller N Kelly R Newell J Kerin MJ. Systemic miRNA-195 differentiates breast cancer from other malignancies and is a potential biomarker for detecting noninvasive and early stage disease. Oncologist. (2010) 15:673. 10.1634/theoncologist.2010-0103 20576643 PMC3228012

[42] Chen W Wang H Chen H Liu S Lu H Kong D. Clinical significance and detection of micro RNA-21 in serum of patients with diffuse large B-cell lymphoma in Chinese population. Eur J Haematol. (2014) 92:407–12. 10.1111/ejh.12263 24400911

[43] Moussay E Wang K Cho J-H van Moer K Pierson S Paggetti J. MicroRNA as biomarkers and regulators in B-cell chronic lymphocytic leukemia. PNAS. (2011) 108:6573–8. 10.1073/pnas.1019557108 21460253 PMC3081030

[44] Li L-M Hu Z-B Zhou Z-X Chen X Liu F-Y Zhang J-F. Serum microRNA profiles serve as novel biomarkers for HBV infection and diagnosis of HBV-positive hepatocarcinoma. Cancer Res. (2010) 70:9798–807. 10.1158/0008-5472.CAN-10-1001 21098710

[45] He Y Ding Y Liang B Lin J Kim T-K Yu H. A systematic study of dysregulated microRNA in type 2 diabetes mellitus. Int J Mol Sci. (2017) 18:456. 10.3390/ijms18030456 28264477 PMC5372489

[46] Moridikia A Mirzaei H Sahebkar A Salimian J. MicroRNAs: potential candidates for diagnosis and treatment of colorectal cancer. J Cell Physiol. (2018) 233:901–13. 10.1002/jcp.25801 28092102

[47] Sun Y Gui H Li Q Luo ZM Zheng MJ Duan JL. MicroRNA-124 protects neurons against apoptosis in cerebral ischemic stroke. CNS Neurosci Ther. (2013) 19:813–9. 10.1111/cns.12142 23826665 PMC6493643

[48] Chen C Hu Q Yan J Yang X Shi X Lei J. Early inhibition of HIF-1α with small interfering RNA reduces ischemic–reperfused brain injury in rats. Neurobiol Dis. (2009) 33:509–17. 10.1016/j.nbd.2008.12.010 19166937

[49] Sepramaniam S Tan J-R Tan K-S DeSilva DA Tavintharan S Woon F-P. Circulating microRNAs as biomarkers of acute stroke. Int J Mol Sci. (2014) 15:1418–32. 10.3390/ijms15011418 24447930 PMC3907877

[50] Fernandez-Valverde SL Taft RJ Mattick JS. MicroRNAs in β-cell biology, insulin resistance, diabetes and its complications. Diabetes. (2011) 60:1825–31. 10.2337/db11-0171 21709277 PMC3121441

[51] Andrews KT Fisher G Skinner-Adams TS. Drug repurposing and human parasitic protozoan diseases. Int J Parasitol Drugs Drug Resist. (2014) 4:95–111. 10.1016/j.ijpddr.2014.02.002 25057459 PMC4095053

[52] Raissi V Zibaei M Raiesi O Samani Z Yarahmadi M Etemadi S. Parasite-derived microRNAs as a diagnostic biomarker: potential roles, characteristics, and limitations. J Parasitic Dis. (2021) 45:546–56. 10.1007/s12639-021-01395-w 34295053 PMC8254842

[53] Schmidt CQ Kennedy AT Tham W-H. More than just immune evasion: hijacking complement by *Plasmodium falciparum. Molecul Immunol*. (2015) 67:71–84. 10.1016/j.molimm.2015.03.006 25816986

[54] Chamnanchanunt S Kuroki C Desakorn V Enomoto M Thanachartwet V Sahassananda D. Downregulation of plasma miR-451 and miR-16 in Plasmodium vivax infection. Exp Parasitol. (2015) 155:19–25. 10.1016/j.exppara.2015.04.013 25913668

[55] Baro B Deroost K Raiol T Brito M Almeida ACG de Menezes-Neto A. Plasmodium Vivax Gametocytes in the Bone Marrow of an Acute Malaria Patient and Changes in the Erythroid miRNA Profile. San Francisco, CA: Public Library of Science (2017). 10.1371/journal.pntd.0005365 PMC5383020 28384192

[56] Hashemi N Sharifi M Tolouei S Hashemi M Hashemi C Hejazi SH. Expression of hsa Let-7a MicroRNA of macrophages infected by *Leishmania major. Int J Med Sci Public Health*. (2018) 5:27–32.

[57] Geraci NS Tan JC McDowell MA. Characterization of micro RNA expression profiles in Leishmaniaâ infected human phagocytes. Parasite Immunol. (2015) 37:43–51. 10.1111/pim.12156 25376316 PMC4287219

[58] Hotez PJ Brindley PJ Bethony JM King CH Pearce EJ Jacobson J. Helminth infections: the great neglected tropical diseases. J Clin Investig. (2008) 118:1311–21. 10.1172/JCI34261 18382743 PMC2276811

[59] Kim Y-K. Extracellular microRNAs as biomarkers in human disease. Chonnam Med J. (2015) 51:51–7. 10.4068/cmj.2015.51.2.51 26306299 PMC4543150

[60] Zibaei M. Parasitic Infections and MicroRNAs. Karaj: Alborz University of Medical Sciences (2018), p. 55. 10.15171/ijep.2018.15

[61] Britton C Winter AD Gillan V Devaney E. microRNAs of parasitic helminths–Identification, characterization and potential as drug targets. Int J Parasitol Drugs Drug Resist. (2014) 4:85–94. 10.1016/j.ijpddr.2014.03.001 25057458 PMC4095049

[62] Lee RC Feinbaum RL Ambros V. The *C. elegans* heterochronic gene lin-4 encodes small RNAs with antisense complementarity to lin-14. Cell. (1993) 75:843–54. 8252621 10.1016/0092-8674(93)90529-y

[63] Poole CB Davis PJ Jin J McReynolds LA. Cloning and bioinformatic identification of small RNAs in the filarial nematode, *B. malayi. Mol Biochem Parasitol*. (2010) 169:87–94. 10.1016/j.molbiopara.2009.10.004 19874857

[64] Xue X Sun J Zhang Q Wang Z Huang Y Pan W. Identification and characterization of novel microRNAs from *S. japonicum. PLoS ONE*. (2008) 3:e4034. 10.1371/journal.pone.0004034 19107204 PMC2603315

[65] de Souza Gomes M Muniyappa MK Carvalho SG Guerra-Sá R Spillane C. Genome-wide identification of novel microRNAs and their target genes in the human parasite *S. mansoni. Genomics*. (2011) 98:96–111. 10.1016/j.ygeno.2011.05.007 21640815

[66] Xu M-J Ai L Fu J-H Nisbet AJ Liu Q-Y Chen M-X. Comparative characterization of microRNAs from the liver flukes *F. gigantica* and *F. hepatica. PLoS ONE*. (2012) 7:e53387. 10.1371/journal.pone.0053387 23300925 PMC3534066

[67] Xu M-J Wang C-R Huang S-Y Fu J-H Zhou D-H Chang Q-C. Identification and characterization of microRNAs in the pancreatic fluke *E. pancreaticum. Parasit Vectors*. (2013) 6:25. 10.1186/1756-3305-6-25 23351883 PMC3621695

[68] Mu Y Cai P Olveda RM Ross AG Olveda DU McManus DP. Parasite-derived circulating microRNAs as biomarkers for the detection of human *S. japonicum* infection. Parasitology. (2019) 147:1–21. 10.1017/S0031182019001690 31840631 PMC7391863

[69] Bagheri A Khorshid HRK Mowla SJ Mohebbi HA Mohammadian A Yaseri M. Altered miR-223 expression in sputum for diagnosis of non-small cell lung cancer. Avicenna J Med Biotechnol. (2017) 9:189. 29090068 PMC5650736

[70] Cheng G Luo R Hu C Cao J Jin Y. Deep sequencing-based identification of pathogen-specific microRNAs in the plasma of rabbits infected with *S. japonicum. Parasitology*. (2013) 140:1751–61. 10.1017/S0031182013000917 23942009

[71] Cai P Gobert GN You H Duke M McManus DP. Circulating miRNAs: potential novel biomarkers for hepatopathology progression and diagnosis of schistosomiasis japonica in two murine models. PLoS Negl Trop Dis. (2015) 9:e0003965. 10.1371/journal.pntd.0003965 26230095 PMC4521869

[72] He X Sai X Chen C Zhang Y Xu X Zhang D. Host serum miR-223 is a potential new biomarker for *S. japonicum* infection and the response to chemotherapy. Parasit Vectors. (2013) 6:272. 10.1186/1756-3305-6-272 24330517 PMC3856452

[73] Burke ML McManus DP Ramm GA Duke M Li Y Jones MK. Temporal expression of chemokines dictates the hepatic inflammatory infiltrate in a murine model of schistosomiasis. PLoS Negl Trop Dis. (2010) 4:e598. 10.1371/journal.pntd.0000598 20161726 PMC2817718

[74] Meningher T Lerman G Regev-Rudzki N Gold D Ben-Dov IZ Sidi Y. Schistosomal MicroRNAs isolated from extracellular vesicles in sera of infected patients: a new tool for diagnosis and follow-up of human schistosomiasis. J Infect Dis. (2016) 215:378–86. 10.1093/infdis/jiw539 28362903

[75] Hoy AM Lundie RJ Ivens A Quintana JF Nausch N. Forster T, et al. Parasite-derived microRNAs in host serum as novel biomarkers of helminth infection. PLoS Negl Trop Dis. (2014) 8:e2701. 10.1371/journal.pntd.0002701 24587461 PMC3930507

[76] Guo X Guo A. Profiling circulating microRNAs in serum of *F. gigantica-*infected buffalo. Mol Biochem Parasitol. (2019) 232:111201. 10.1016/j.molbiopara.2019.111201 31377228

[77] Tritten L Burkman E Moorhead A Satti M Geary J Mackenzie C. Detection of circulating parasite-derived microRNAs in filarial infections. PLoS Negl Trop Dis. (2014) 8:e2971. 10.1371/journal.pntd.0002971 25033073 PMC4102413

[78] Quintana JF Makepeace BL Babayan SA Ivens A Pfarr KM Blaxter M. Extracellular Onchocerca-derived small RNAs in host nodules and blood. Parasit Vectors. (2015) 8:58. 10.1186/s13071-015-0656-1 25623184 PMC4316651

[79] Tritten L O'Neill M Nutting C Wanji S Njouendoui A Fombad F. *Loa loa* and *Onchocerca ochengi* miRNAs detected in host circulation. Mol Biochem Parasitol. (2014) 198:14–7. 10.1016/j.molbiopara.2014.11.001 25461483

[80] Silakit R Loilome W Yongvanit P Chusorn P Techasen A Boonmars T. Circulating mi R-192 in liver fluke-associated cholangiocarcinoma patients: a prospective prognostic indicator. J Hepatobiliary Pancreat Sci. (2014) 21:864–72. 10.1002/jhbp.145 25131257

[81] Plieskatt J Rinaldi G Feng Y Peng J Easley S Jia X. A microRNA profile associated with *O. viverrini*-induced cholangiocarcinoma in tissue and plasma. BMC Cancer. (2015) 15:309. 10.1186/s12885-015-1270-5 25903557 PMC4417245

[82] Chen X Li Z-Y Maleewong W Maleewong P Liang J Zeng X. Serum aca-mir-146a is a potential biomarker for early diagnosis of *An. cantonensis* infection. Parasitol Res. (2014)113:3221–7. 10.1007/s00436-014-3984-8 24951166

[83] Guo X Zheng Y. Expression profiling of circulating miRNAs in mouse serum in response to *E. multilocularis* infection. Parasitology. (2017) 144:1079–87. 10.1017/S0031182017000300 28270244

[84] Alizadeh Z Mahami-Oskouei M Spotin A Kazemi T Ahmadpour E Cai P. Parasite-derived microRNAs in plasma as novel promising biomarkers for the early detection of hydatid cyst infection and post-surgery follow-up. Acta Trop. (2020) 202:105255. 10.1016/j.actatropica.2019.105255 31682814

[85] Cai P Mu Y Olveda RM Ross AG Olveda DU McManus DP. Circulating miRNAs as footprints for liver fibrosis grading in schistosomiasis. EBioMedicine. (2018) 37:334–43. 10.1016/j.ebiom.2018.10.048 30482723 PMC6286190

[86] Lagatie O Debrah LB Debrah A Stuyver LJ. Plasma-derived parasitic microRNAs have insufficient concentrations to be used as diagnostic biomarker for detection of *Onchocerca volvulus* infection or treatment monitoring using LNA-based RT-qPCR. Parasitol Res. (2017) 116:1013–22. 10.1007/s00436-017-5382-5 28111713 PMC5313568

[87] Buck AH Coakley G Simbari F McSorley HJ Quintana JF Le Bihan T. Exosomes secreted by nematode parasites transfer small RNAs to mammalian cells and modulate innate immunity. Nat Commun. (2014) 5:5488. 10.1038/ncomms6488 25421927 PMC4263141

[88] Zhu L Dao J Du X Li H Lu K Liu J. Altered levels of circulating miRNAs are associated *S. japonicum* infection in mice. Parasit Vectors. (2015) 8:196. 10.1186/s13071-015-0806-5 25885182 PMC4391475

[89] Cheng G. Circulating miRNAs: roles in cancer diagnosis, prognosis and therapy. Adv Drug Deliv Rev. (2015) 81:75–93. 10.1016/j.addr.2014.09.001 25220354

[90] Regev-Rudzki N Wilson DW Carvalho TG Sisquella X Coleman BM Rug M. Cell–cell communication between malaria-infected red blood cells *via* exosome-like vesicles. Cell. (2013) 153:1120–33. 10.1016/j.cell.2013.04.029 23683579

[91] Rekker K Saare M Roost AM Kubo A-L Zarovni N Chiesi A. Comparison of serum exosome isolation methods for microRNA profiling. Clin Biochem. (2014) 47:135–8. 10.1016/j.clinbiochem.2013.10.020 24183884

[92] Logozzi M Mizzoni D Di Raimo R Fais S. Exosomes: a source for new and old biomarkers in cancer. Cancers. (2020) 12:2566. 10.3390/cancers12092566 32916840 PMC7565506

[93] Silesâ Lucas AM Morchon R Simon F Manzanoâ Roman R. Exosomeâ transported micro RNA s of helminth origin: new tools for allergic and autoimmune diseases therapy. Parasite Immunol. (2015) 37:208–14. 10.1111/pim.12182 25712154

[94] Nowacki FC Swain MT Klychnikov OI Niazi U Ivens A Quintana JF. Protein and small non-coding RNA-enriched extracellular vesicles are released by the pathogenic blood fluke *S. mansoni. J Extracell Vesicles*. (2015) 4:28665. 10.3402/jev.v4.28665 26443722 PMC4595467

[95] Wang L Li Z Shen J Liu Z Liang J Wu X. Exosome-like vesicles derived by *S. japonicum* adult worms mediates M1 type immune-activity of macrophage. Parasitol Res. (2015) 114:1865–73. 10.1007/s00436-015-4373-7 25855345

[96] Marcilla A Trelis M Cortés A Sotillo J Cantalapiedra F Minguez MT. Extracellular vesicles from parasitic helminths contain specific excretory/secretory proteins and are internalized in intestinal host cells. PLoS ONE. (2012) 7:e45974. 10.1371/journal.pone.0045974 23029346 PMC3454434

[97] Fromm B Trelis M Hackenberg M Cantalapiedra F Bernal D Marcilla A. The revised microRNA complement of *Fasciola hepatica* reveals a plethora of overlooked microRNAs and evidence for enrichment of immuno-regulatory microRNAs in extracellular vesicles. Int J Parasitol. (2015) 45:697–702. 10.1016/j.ijpara.2015.06.002 26183562

[98] Thakur BK Zhang H Becker A Matei I Huang Y Costa-Silva B. Double-stranded DNA in exosomes: a novel biomarker in cancer detection. Cell Res. (2014) 24:766. 10.1038/cr.2014.44 24710597 PMC4042169

[99] Chaiyadet S Sotillo J Smout M Cantacessi C Jones MK Johnson MS. Carcinogenic liver fluke secretes extracellular vesicles that promote cholangiocytes to adopt a tumorigenic phenotype. J Infect Dis. (2015) 212:1636–45. 10.1093/infdis/jiv291 25985904 PMC4621255

[100] Bernal D Trelis M Montaner S Cantalapiedra F Galiano A Hackenberg M. Surface analysis of *Dicrocoelium dendriticum*. The molecular characterization of exosomes reveals the presence of miRNAs. J Proteom. (2014)105:232–41. 10.1016/j.jprot.2014.02.012 24561797

[101] Ancarola ME Marcilla A Herz M Macchiaroli N Pérez M Asurmendi S. Cestode parasites release extracellular vesicles with microRNAs and immunodiagnostic protein cargo. Int J Parasitol. (2017) 47:675–86. 10.1016/j.ijpara.2017.05.003 28668323

[102] Zhu S Wang S Lin Y Jiang P Cui X Wang X. Release of extracellular vesicles containing small RNAs from the eggs of *S. japonicum. Parasit Vectors*. (2016) 9:574. 10.1186/s13071-016-1845-2 27825390 PMC5101684

[103] Liu J Zhu L Wang J Qiu L Chen Y Davis RE. *Schistosoma japonicum* extracellular vesicle miRNA cargo regulates host macrophage functions facilitating parasitism. PLoS Pathog. (2019) 15:e1007817. 10.1371/journal.ppat.1007817 31163079 PMC6548406

[104] Hansen EP Kringel H Williams AR Nejsum P. Secretion of RNA-containing extracellular vesicles by the porcine whipworm, *Trichuris suis. J Parasitol*. (2015) 101:336–41. 10.1645/14-714.1 25723295

[105] Rangel G Teerawattanapong N Chamnanchanunt S Umemura T Pinyachat A Wanram S. Candidate microRNAs as biomarkers in malaria infection: a systematic review. Curr Mol Med. (2020) 20:36–43. 10.2174/1566524019666190820124827 31429687

[106] Sempere LF Freemantle S Pitha-Rowe I Moss E Dmitrovsky E Ambros V. Expression profiling of mammalian microRNAs uncovers a subset of brain-expressed microRNAs with possible roles in murine and human neuronal differentiation. Genome Biol. (2004) 5:1–11. 10.1186/gb-2004-5-3-r13 15003116 PMC395763

[107] Kloosterman WP Wienholds E de Bruijn E Kauppinen S Plasterk RHA. *In situ* detection of miRNAs in animal embryos using LNA-modified oligonucleotide probes. Nat Methods. (2006) 3:27–9. 10.1038/nmeth843 16369549

[108] Wang J Chen J Chang P LeBlanc A Li D Abbruzzesse JL. MicroRNAs in plasma of pancreatic ductal adenocarcinoma patients as novel blood-based biomarkers of DiseasePlasma MicroRNAs in pancreatic cancer. Cancer Prev Res. (2009) 2:807–13. 10.1158/1940-6207.CAPR-09-0094 19723895 PMC5859193

[109] Wang J Raimondo M Guha S Chen J Diao L Dong X. Circulating microRNAs in pancreatic juice as candidate biomarkers of pancreatic cancer. J Cancer. (2014) 5:696. 10.7150/jca.10094 25258651 PMC4174514

[110] Wang J Paris PL Chen J Ngo V Yao H Frazier ML. Next generation sequencing of pancreatic cyst fluid microRNAs from low grade-benign and high grade-invasive lesions. Cancer Lett. (2015) 356:404–9. 10.1016/j.canlet.2014.09.029 25304377 PMC6200344

[111] Mekonnen GG Pearson M Loukas A Sotillo J. Extracellular vesicles from parasitic helminths and their potential utility as vaccines. Exp Rev Vaccines. (2018) 17:197–205. 10.1080/14760584.2018.1431125 29353519

[112] Mendona LSO Santos JM Kaneto CM de Carvalho LD Lima-Santos J Augusto DG. Characterization of serum cytokines and circulating microRNAs that are predicted to regulate inflammasome genes in cutaneous leishmaniasis patients. Exp Parasitol. (2020) 210:107846. 10.1016/j.exppara.2020.107846 32001303

[113] Sanprasert V Kerdkaew R Srirungruang S Charuchaibovorn S Phadungsaksawasdi K Nuchprayoon S. Development of conventional multiplex PCR: a rapid technique for simultaneous detection of soil-transmitted helminths. Pathogens. (2019) 8:152. 10.3390/pathogens8030152 31527459 PMC6789620

